# Going back into the wild: the behavioural effects of raising sea urchins in captivity

**DOI:** 10.1093/conphys/coaa015

**Published:** 2020-04-04

**Authors:** G Brundu, S Farina, P Domenici

**Affiliations:** 1 IMC-International Marine Centre, Loc. Sa Mardini, Torre Grande, 09170 Oristano, Italy; 2 IAS- Institute of Anthropic Impact and Sustainability in Marine Environment, CNR, Loc. Sa Mardini, Torre Grande, 09170 Oristano, Italy

**Keywords:** animal behaviour, captivity, locomotion, repopulation, rewilding, sea urchins

## Abstract

Sea urchin harvesting has rapidly expanded in the last decades. Since many sea urchin species play important ecological role, large-scale commercial sea urchin fisheries can have complex effects on benthic communities. In many temperate regions, overharvesting has compromised marine ecosystems to such an extent that reintroduction of sea urchins raised in captivity may be a valid solution for the enhancement of depleted marine wild populations. In some regions of the Mediterranean Sea, improving the growth efficiency of captive sea urchin *Paracentrotus lividus* to be reintroduced has become a widespread practice. However, no study has yet considered the potential behavioural effects of raising sea urchins in captivity when they are introduced in the natural environment. This study provides information about the behavioural effects of captivity on *P. lividus* in terms of locomotion performance, a trait that can be fundamental for responding to predators and for relocation after environmental disturbances such as currents and waves. Movements of captive-born and wild sea urchins were video-recorded and compared in (i) total exposure to external cues, (ii) partial exposure to external cues and (iii) absence of external cues. Latency of locomotion, average speed and average velocity of sea urchins showed significant differences with respect to the level of exposure and their origin (i.e. wild vs. captive-born). Our results demonstrate that captive-born sea urchins in the wild showed long latency and slower locomotor performance when compared to wild sea urchins. Conversely, the straightness-of-path and locomotion direction of captive-born and wild sea urchins were similar in natural settings. Our results therefore suggest that captive-born sea urchins suffer the negative effects of captivity when introduced in a natural environment. Understanding the factors that decrease the performance of sea urchin will be important for developing procedures aimed at minimizing the negative effect of captivity before release into the wild.

## Introduction

The overexploitation of species involved in the typical tri-trophic interaction ‘fish-sea urchins-macrophyte’ is one of the clearest examples reported for coastal ecosystems of temperate regions (Sala *et al*., 1998; Loi *et al*., 2017). Overfishing is known to have led to the uncontrolled proliferation of herbivore sea urchins, such as the functional key species *Paracentrotus lividus* in Mediterranean Sea, with dramatic consequences on benthic communities (McClanahan and Sala, 1997; Sala *et al*., 1998; Steneck *et al*., 2002; Steneck *et al*., 2004; Guidetti and Sala, 2007; Prado *et al*., 2007; Sala *et al*., 2012). The decline of the natural fish stocks caused invertebrate to be part of the global fishery (Conand and Byrne 1993; Anderson *et al*., 2011), for example the sea urchin harvesting of *P. lividus* in the South of France and Italy (Andrew *et al*., 2002; Pais *et al*., 2012).

In general, the consequence of the systematic removal of sea urchin is that community structures have largely changed in several temperate macrophyte ecosystems (e.g. McShane *et al*., 1994; Villouta, 2000; Villouta *et al*., 2001). In temperate reefs, large-scale harvesting of sea urchins can determine a rapid development of stands of large brown algae and consequent changes in community composition (Andrew *et al*., 2002). Thus, along with climate change, overfishing in general has compromised marine ecosystems to such an extent that many traditional conservation targets are now out of reach like the case of the Bay of Biscay anchovy (Barange *et al.*, 2010; Rosenberg and Fong, 2013).

In the context of resource over exploitation, like in many terrestrial ecosystems (Ripple and Beschta, 2012), the reintroduction of organisms raised in captivity is considered as a potential solution for the enhancement of depleted marine wild populations (Juinio-Meñez *et al*. 2008; Cárcamo, 2015; Couvray *et al*., 2015). For example, a number of reintroductions have been obtained on threatened populations of commercial fish species (Hutchison *et al*., 2012). However, it is well known that captivity can cause significant alterations in behavioural traits (Brooker *et al*., 2016). Captive-born fish may be less responsive to natural predators (Alvarez and Nicieza, 2003), may fail to recognize natural or wild foods or may behave differently than wild fish. Specifically, they may not use available shelter, they may bury less and may form less cohesive shoals than wild conspecifics (Hutchison *et al*., 2012).

Captive breeding has also been used to produce and reintroduce sea urchins in the wild when populations are locally threatened by intense harvesting (Brady and Scheilbling, 2005; Meñez, 2016; Hannon, 2018). Many studies have been carried out to improve the efficiency of breeding in terms of growth of organisms and their successive reintroduction into the wild, but no significant results have been reached so far (Le Gall and Bucaille, 1989; Le Gall, 1990; Grosjean, *et al*., 1998; Kelly *et al*., 1998; Robinson and Colborne, 1998; Cook and Kelly, 2007). Reintroduction of captive *P. lividus* was found to return the stock abundance back to the original density, to restore the reproductive capacity of depleted populations (Juinio-Meñez *et al*., 2008; Loi *et al*., 2017), and it is supposed to be a restoration action that returns the ecosystem back to the way it was (e.g. Corlett, 2016).

Until now, however, no study has considered the potential behavioural effects of raising of sea urchins in captivity, once they are released into the wild. Maladaptive behaviours may be fatal for the success of sea urchin reintroduction, as they are largely preyed upon by fish through the top-down control mechanism in marine benthic ecosystems (McClanahan and Sala, 1997; Guidetti *et al*., 2004; Hereu *et al*., 2008). Survival strongly depends on the ability of sea urchins to migrate among exposed feeding areas and available shelters provided by the habitat structure such as rocky crevices, holes or seagrass coverage (e.g. Hereu *et al*., 2005; Farina *et al*., 2009; Farina *et al*., 2014). Thus, the prowess to feed, their locomotor performance and their anti-predator behaviours become crucial points for the long-term success of the reintroduction.

In general, sea urchin breeding does not include a prior acclimation before the reintroduction into the wild (Shier, 2016). Captive-born specimens are bred in confined spaces at high-density conditions which makes their movements difficult. Individuals are fed several times a week and in absence of predatory threats, in order to speed up the growth until a suitable size for reintroduction is reached (see methods). Thus, in order to correct for potential behavioural malfunctions due to captivity, we need to increase our understanding of how captive-born sea urchins behave in the wild environment. It is known that many factors such as grazing availability, predation risk (Mattisson *et al*., 1977; Shepherds and Boudouresque, 1979; Jones and Andrew, 1990; Alvarez and Nicieza, 2003), currents and waves (Lissner, 1980; James, 2000; Hutchison *et al*., 2012; Cohen-Rengifo *et al*., 2018), substrate (Laur *et al*., 1986) and topography (Rodriguez and Ojeda, 1998) can affect sea urchin behaviour. For instance, Dumont *et al*. (2007) reported that sea urchins move in random directions most of the time until they detect the food source. Numerous studies report a direct effect of hydrodynamics on the locomotion of sea urchins and other echinoderm species. The movement rate of *Strongylocentrotus droebachiensis* (Dumont *et al*., 2006) decreases with increasing current speed. *Echinometra oblonga* and *Echinometra mathaei* generally move along the water flow and rarely across the current (Pan *et al*., 2015). In addition, environmental cues, such as sunlight, food sources, shelters and water circulation, strongly influence sea urchin movements (e.g. Yoshida, 1966; Dance, 1987; Domenici *et al*., 2003).

The aim of the current study is to evaluate potential behavioural malfunctions and locomotor performance in captive-born *P. lividus* as a first step towards evaluating their suitability for reintroduction into the wild. In order to test different levels of environmental exposure, observations are carried out in the field (open and closed exposures) and in the laboratory (indoor exposure).

## Materials and methods

### Origin of the animals

Captive-born juveniles of *P. lividus* (hereafter Captive) were originated from 10 mixed-broodstock adult sea urchins (diameter > 4.5 cm), consisting of five male and five female. Broodstock were collected from 5-m depth at Su Pallosu, Sardinia, Italy (40°03′205″ N, 8°24′794″ E). Methods for reproduction and larval and post-larval rearing followed those used by Brundu *et al*. (2016, 2017).

**Figure 1:**
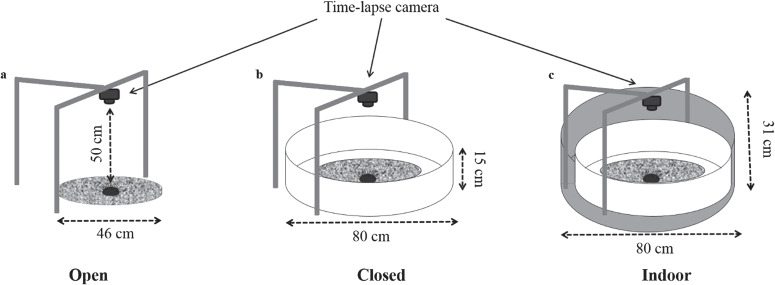
Experimental set-up of three treatments with different levels of environmental exposure: Open (**a**); Closed (**b**); Indoor (**c**)

Both larvae and juveniles were reared in the International Marine Centre—IMC laboratories (Oristano, Sardinia, Italy) under controlled conditions in filtered (1 μm) natural seawater at 36.5 ± 1 ppt, temperature of 20 ± 2°C, and exposed to a 12 h L/12 h D photoperiod. Juvenile individuals were maintained in a recirculating aquaculture system (flow rate of 5 L min^−1^) consisting of a 600-L rectangular tank and supplied with biological and mechanical filtration (10 μm). Individuals were fed *ad libitum* with fresh thalli of *Ulva lactuca* for about 16 months, in order to optimize their growth until they reached the suitable size to be reintroduced (about 1.5 cm of a test diameter without spines; Tegner, 1989). Moreover, wild juveniles (hereafter Wild) in the same range of size were collected randomly by scuba diving from a population located on a bedrock in Su Pallosu bay between 1 and 2 m depth (Western Sardinia; 40°03′103″ N, 8°24′241″ E).

Before starting the observations, both in the field and in laboratory, captive-born and wild sea urchins were acclimated for 24 h in standard conditions of no food and at 20°C temperature to the environment of the experiment. Acclimation indoor was carried out in circular tanks of white resin of 50-L capacity, while in the sea, urchins were placed in nylon nets and left on the bottom in the area where the experiments were going to take place.

### Locomotor performance

#### Conditions of exposure to external cues

Locomotor performance of captive and wild sea urchins was evaluated under three different treatment conditions of exposure to external cues: (i) total exposure to external cues (Open), (ii) partial exposure to external cues (Closed) that were verified in the field and (iii) absence of external cues (Indoor).

For each treatment, sea urchins were placed one by one in the middle of the field of recording that consisted in a circular platform of flat granite rock (46 cm diameter and 4 cm thickness).

Observations were carried out in the summer time, when the temperature and salinity of the seawater were similar to the laboratory tank (see below).

For the Open exposure, the open field condition of Su Pallosu bay at 1-m depth, characterized by rocky seabed with erect and turf coverage, primarily *Cystoseira* spp., *Dictyota* spp., *Padina pavonica* and *Jania rubens* were used (Loi *et al*., 2017; Fig. 1a).

The Closed exposure was set up at the same field site, covering the seabed with a circular surface of white-plastic cloth of 80 cm diameter. A white plastic ring of 15-cm height was positioned along the perimeter of the cloth, standardizing the horizontal view of the sea urchin (see Fig. 1b).

The Indoor treatment was set up indoor in a cylindrical tank (157 L volume, 80 cm diameter, 31 cm depth) coated on the inner side with a white plastic cloth to a height of 15 cm. Seawater was at a salinity of 37.5 ppt and temperature at 20 ± 2°C in static conditions, with no aeration and no direct light sources (Fig. 1c).

#### Video recording

Video trials to record locomotion performance were conducted over a 3-day period between 09:30 and 11:00 am. The study was carried out during typical summer time condition characterized by high atmospheric pressure, absence of wind and calm water (Farina *et al*., 2018).

Prior to the experiments, both Captive and Wild were acclimated and maintained without food for 24 h. For each treatment, from 10 to 16 individuals from each group (Captive and Wild) were measured. Before each video recording, the platform was carefully cleaned and overturned to avoid possible influences of the previous test.

**Table 1:** Summary of variables describing locomotion performance

**Type**	**Expo**	**N° obs.**	**Latency (s)**	**SE**	**Average speed (cm/min)**	**SE**	**Average velocity (cm/min)**	**SE**	**Straight**	**SE**
Wild	Open	10	0.5	0.5	6.0	1.8	5.4	5.1	0.9	0.1
Captive	Open	13	3.5	1.6	4.6	1.0	3.8	3.4	0.8	0.2
Wild	Closed	16	12.8	3.4	6.3	1.2	4.8	2.4	0.8	0.2
Captive	Closed	12	22.1	4.4	5.5	0.7	4.3	3.3	0.8	0.2
Wild	Indoor	15	45.3	5.5	2.3	0.5	1.2	1.8	0.6	0.3
Captive	Indoor	15	6.0	1.7	4.2	1.5	2.6	4.5	0.6	0.2

Number of observations and mean values with standard error of latency, average speed, average velocity and straightness-of-path in relation of the Origin of the animals and level of the exposure to the environmental cues.

Sea urchin movements were recorded with high-definition action cameras (GoPro Hero®) with time-lapse set using a framing rate of 12 frames per minute. The camera was positioned 50 cm above the circular platform (see Fig. 1). For each trial, recording started as soon as the individual was positioned on the bottom and ended when the individual reached the edge of the circular platform, or for a maximum period of 15 min if individuals had not reached the edge of the platform within this time period.

#### Variables describing locomotion performance

Performance of sea urchin locomotion was assessed by estimating the following variables: (i) latency of locomotion, (ii) average speed, (iii) average velocity, (iv) straightness-of-path and (v) locomotion direction (Table 1). The video images representing the successive positions occupied by the individual every 5 s were analysed with *WINanalyze 1.5 software* (Mikromak®).

**Figure 2:**
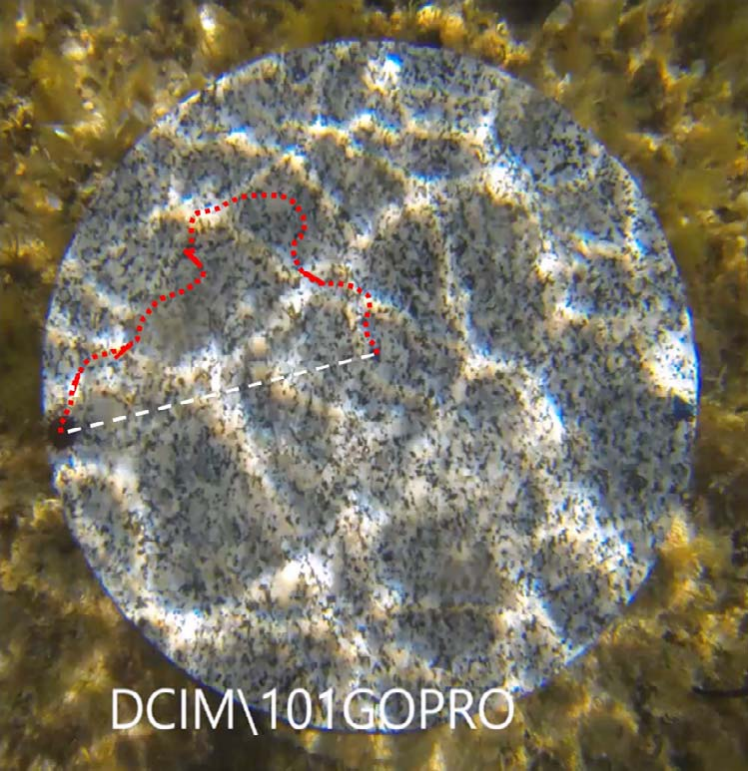
An example of a track of a Captive-born sea urchin. The red dotted line represents the position of the individual in each frame while the white dotted line is the linear distance between the position of the individual in the first and the last frame.

(i) Latency of locomotion was measured as the time (seconds) between the beginning of the trial (i.e. Frame 0, when the sea urchin was positioned at the centre of the circular platform) and the first movement of the sea urchin which corresponded to distancing at least 3 mm from the starting point. This variable therefore measures the readiness to move after repositioning which can be an important trait, for example when sea urchins are dislocated.

(ii) Average speed was measured from the first frame in which an individual moved (cm/min), i.e. after the time corresponding to latency of locomotion until the individual reached the edge of the circular platform, or for a maximum period of 15 min if individuals had not reached the edge of the platform within this time period. Spatial positions (*x* and *y* coordinates) were determined from successive frames over the duration of the recording period (i.e. until the sea urchin reached the edge of the circular platform) (Fig. 2). Spatial coordinates were converted to distance travelled (cm) to calculate individual average speed (i.e. independent of direction) throughout each trial. Average speed is therefore a measure of the mobility with which a sea urchin can displace itself through crawling in any direction.

(iii) Average velocity (i.e. in the direction of travel) was calculated as *Ld/t*, where *Ld* represents the straight segment between the position of the individual in the first (Frame 0) and the last frame (Fig. 2), and *t* is the time taken to cover this distance (cm/min). Average velocity is therefore related to the combination of the ability of the sea urchins to move in a straight direction and at a fast speed of locomotion, and it includes the delay due to latency of locomotion. For a given average speed, average velocity is higher when the path of locomotion is straight rather than convoluted (see Fig. 2).

(iv) Straightness-of-path (*I*) is a dimensionless index, calculated as *I* = *D*/*L* (Batschelet, 1981), where *D* corresponds to the distance between the positions of an individual in the first and last frame, and *L* is the sum of all segments based on the positions in successive frames (length of path). The I index ranges from 0, corresponding to maximum tortuosity, to 1, corresponding to maximum linearity. This variable is therefore related to the ability of sea urchins to move in a straight path.

**Figure 3:**
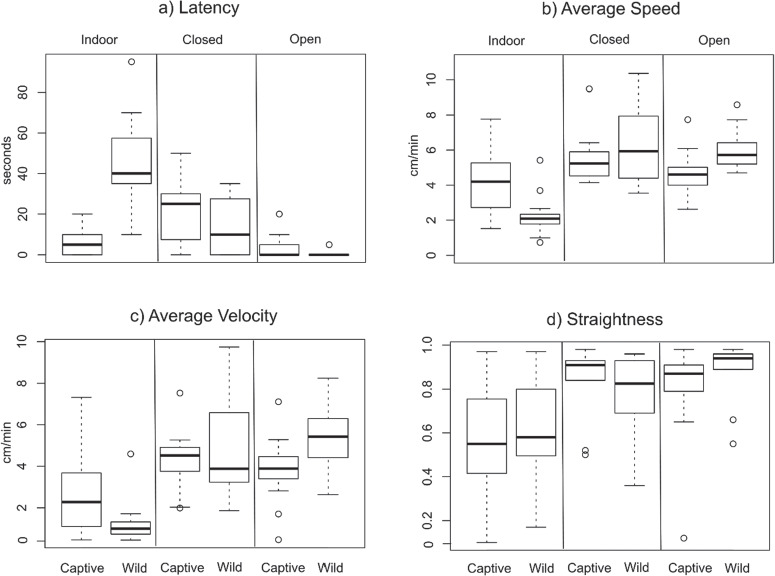
Box plots represent (**a**) latency of locomotion (sec), (**b**) average speed (cm/min), (**c**) average velocity (cm/min) and (**d**) straightness-of-path, for Captive and Wild juveniles in Indoor, Closed and Open treatments. Values are expressed as mean ± SE (see Table 1). Significant differences are reported in Table 2 and Appendix 1.

(v) Locomotion direction (0° to 360°) was determined as the line connecting the first and last frame positions of the trial, with 0° being North, 180° South, 90° East and 270° West. This is a circular variable (Batschelet, 1981), related to the direction undertaken by the sea urchin.

### Data analysis

We carried out the analysis of variance for latency, average speed, average velocity and straightness-of-path. The analysis was set with ‘Origin’ (Captive and Wild) and ‘Exposure’ (Indoor, Open and Closed) as fixed factors, while ‘Size’ of specimens as random factor. Least-squares means were conducted as multiple comparisons among interactions of factors (Russell, 2016). Assumptions of normal distribution and homogeneity of variance of the response variables were analysed using D’Agostino-Pearson and Cochran’s tests. Average speed and average velocity that follow normal distribution but whose replicates are unbalanced were analysed with the linear mixed-effects model for repeated-measures ANOVA two-ways and random factor. In contrast, given the non-normal distribution followed by latency and straightness-of-path, analysis of variance was performed after an alignment and ranking step with the analysis of Aligned Rank Transform with random factor (Art; Wobbrock *et al*., 2011). All the analyses were performed using R Studio (R v. 3.1.0, Development Core Team, 2014).

Locomotion direction was treated as a circular variable and was analysed for randomness using Rayleigh’s test (Batschelet, 1981). Two-way ANOVA for circular data (based on Harrison and Kanji, 1988; Berens, 2009) and post hoc multiple comparisons based on a circular statistics (Mardia–Watson–Wheeler test; Batschelet, 1981) were run to test the effect of ‘Origin’, ‘Exposure’ and the interaction ‘Origin’^*^‘Exposure’.

## Results

Locomotor performance in general was affected by the Origin of the sea urchins, by their Exposure to the environmental cues and by the interactions of these two factors. Captive had on average a significantly different latency than Wild (Fig. 3a, Table 2a and Supplementary Fig. 1a). Specifically, latency of Captive was seven times longer than that of Wild in the Open exposure (3.5 ± 1.6 s versus 0.5 ± 0.5 s respectively) but 7.5 times shorter in the Indoor exposure (6.0 ± 1.7 s versus 45.3 ± 5.5 s respectively). Latency in the Closed exposure resulted also significantly different between the Origins (22.1 ± 4.4 s for Captive and 12.8 ± 3.4 s for Wild).

**Table 2:** Two-way ANOVA for locomotion performance variables

**a.** Latency			
*Source*	*Numerator df*	*F value*	*P value*
Origin	1	19.058	**<0.001**
Exposure	2	29.889	**<0.001**
Origin*Exposure	2	32.685	**<0.001**
**b.** Average speed			
*Source*	*Numerator df*	*F value*	*P value*
Origin	1	7.31179	**0.0086**
Exposure	2	5.55492	**0.0058**
Origin^*^Exposure	2	5.33711	**0.007**
**c.** Average velocity			
*Source*	*Numerator df*	*F value*	*P value*
Origin	1	0.781	**0.0015**
Exposure	2	22.425	0.119
Origin^*^Exposure	2	8.130	**0.0006**
**d.** Straightness-of-path			
*Source*	*Numerator df*	*F value*	*P value*
Origin	1	0.006	0.93836
Exposure	2	11.209	**<0.001**
Origin*Exposure	2	0.667	0.51599

Two-way analysis of variance (ANOVA) for (a) latency, (b) average speed, (c) average velocity and (d) straightness-of-path in relation to the Origin of the animals (two levels), the Exposure (three levels) and their interaction as fixed factors. Degree of freedom (df), *F* value (*F*) and significance level (*P* value) are provided for fixed effects. Significant effects are given in bold.

Average speed was significantly affected by Origin, Exposure and their interaction (Table 2b). While the average speed of Wild was significantly different between Indoor (2.3 ± 0.5 cm/min) and Open exposures (6.0 ± 1.8 cm/min), the average speed of Captive did not differ among exposures (4.2 ± 1.5 cm/min and 4.6 ± 1 cm/min Indoor and in the Open exposure respectively). In Open exposure, average speed was 23% lower in Captive than Wild (Fig. 3b, Table 2b and Supplementary Fig. 1b).

Average velocity was affected by Origin and by the interaction between Origin and Exposure. Wild had a significantly higher average velocity in Open and Closed exposures than Indoor, while in Captive was not significantly different among the three exposures (Fig. 3c, Table 2c and Supplementary Fig. 1c). In Open exposure, the average velocity was 30% lower in Captive (3.8 ± 3.4 cm/min) than Wild (5.4 ± 5.1 cm/min).

Straightness-of-path was significantly affected by the Exposure. Straightness-of-path for both wild and captive sea urchins was lowest in Indoor (0.6 ± 0.3 and 0.6 ± 0.2 respectively) and highest in the Open exposure (0.9 ± 0.1 and 0.8 ± 0.1 respectively, Fig. 3d and Table 2d).

**Figure 4:**
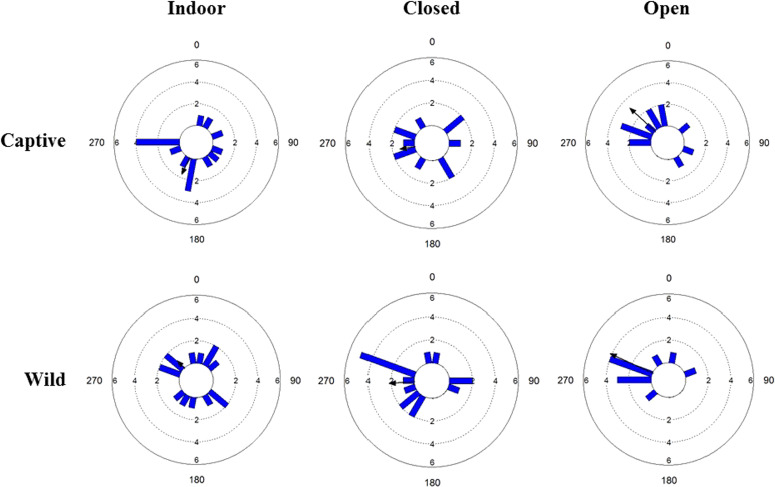
Circular histograms representing frequency of the orientation of crawling trajectories for the two types of sea urchins, Captive and Wild, in the different exposure treatments, Indoor, Closed and Open. 0°, 90°, 180°, 270° represent North, East, South and West, respectively. The black arrow lines indicate the mean orientation, and its length is the mean vector (*r*, from 0 to 1; *r* = 1 is represented by an arrow that reaches the edge of the outer circle). Concentric circles represent the frequency of observations. Bin width is set at 20°.

Locomotion direction was significantly different from a random pattern for both Captive (312.7 ± 64.8 degrees) and Wild (295.6 ± 52.2 degrees) in Open exposure, indicating a tendency for moving towards the North-West direction, while it resulted random in all other cases (see Fig. 4 and Table 3). Exposure was the only Factor that resulted to significantly influence the locomotion direction in the two-way ANOVA for circular data (Table 4). Significant differences were found between Open exposure and Indoor within Captive (see Supplementary Fig. 2).

**Table 3:** Summary results of Rayleigh’s test for locomotion direction

**Origin**	**Exposure**	**N° obs.**	**Mean direction**	**Circular standard deviation**	**Length of mean vector (*r*)**	**Rayleigh test (*Z*)**	**Rayleigh test (*P*)**
Wild	Closed	16	266.7°	80.8°	0.4	2.2	0.111
Captive	Closed	12	259.6°	99.5°	0.2	0.6	0.565
Wild	Open	10	295.6°	52.2°	0.7	4.4	**0.009**
Captive	Open	13	312.7°	64.8°	0.5	3.6	**0.024**
Wild	Indoor	14	295.7°	118.9°	0.1	0.2	0.833
Captive	Indoor	15	205.1°	92°	0.3	1.1	0.325

Tables representing number of observations, mean direction, circular standard deviation, length of mean vector (*r*), Rayleigh test (Z) and Rayleigh test (*P*) in relation to the Origin of the animals and level of the exposure to the environmental cues (Exposure).

**Table 4:** Two-way ANOVA for circular data of locomotion direction

Locomotion direction			
*Source*	*Numerator df*	*Chi^2^*	*P value*
Origin	2	0.4304	0.8064
Exposure	4	10.2398	**0.0366**
Origin^*^Exposure	2	2.2269	0.8358

Two-way analysis of variance (ANOVA) for locomotion direction data, in relation to the Origin of the animals, the level of the exposure to the environmental cues (Exposure) and their interaction. Degree of freedom (numerator df), chi^2^ and significance level (*P* value) are provided for fixed effects.

## Discussion

Our results show that most performance traits of Wild and Captive tended to be opposite in the different environmental exposures. Accordingly, sea urchins kept in captivity for 16 months showed some critical deficits in locomotion performance compared to wild sea urchins when observed in the Open exposure. Specifically, the average velocity was on average 30% lower in Captive than Wild. This was in part due to the longer latency of the Captive, since a slow start (as is the cases for Captive) has a negative effect on the calculated average velocity. The other factor that contributes to a lower average velocity in captive urchin is their lower average speed, because the straightness of path was not different between wild and captive sea urchins.

Although from the perspective of potential repopulation the main aspect of interest is the effect of captivity on sea urchins when introduced in the wild, it is worthwhile noting that wild sea urchins tended to perform better in their habitat of origin. Conversely, the locomotor performance of captive sea urchins did not decrease significantly when they were tested in nature compared to indoor, although it was significantly lower than that of wild sea urchins. Therefore, from a fitness perspective, it can be hypothesized that captive sea urchins would have a selective disadvantage compared to wild ones in natural conditions.

One of the main variables that were affected by captivity was latency of locomotion. The captive-born sea urchins released ‘into the wild’ were seven times slower than wild sea urchins to initiate locomotion (i.e. latency). Ecologically, latency of locomotion could be seen as an indicator of readiness to move after a disturbance. If the readiness to move is low, a captive-born sea urchin could reach a shelter or escape from dangerous situations later than a wild one, and this could potentially have fitness consequences (such as a higher predation rate). This principle may apply to all other variables and particularly to average speed and velocity, since if captive sea urchins are slower than wild ones, they may take more time to reach a shelter and slower to escape from a threat. Recent investigations showed that short-term captivity (i.e. up to 4 days) of red sea urchin *Mesocentrotus franciscanus* affects antipredator behaviours such as self-righting and predator escape responses (Bose *et al*., 2019). Given that wild sea urchins that experienced short captivity showed lower antipredator response after release (Bose *et al*., 2019), a much longer period (such as being raised in captivity) may also result in negative effects in terms of antipredator performance. Similarly, when predation rate was estimated on a pool of captive-born sea urchins in a Marine Protected Area, where predation pressure is supposed to be maximal (Guidetti and Sala, 2007), it was on average 16% higher than for wild sea urchins (Farina and Brundu’s unpublished data). This suggests that chances to survive into the wild of captive-born sea urchins may be lower than of wild sea urchins. However, if one was to assess behaviour a few days after reintroduction, it is possible that the behavioural differences would have decreased. Similarly, a longer period of acclimation may have limited the deficit in performance.

It is widely reported that sea urchins are able to sense external stimuli (e.g. presence of food and predators, environmental cues such as light and shelters) and move in response to them (Garnick, 1978; Scheibling and Hamm, 1991; Domenici *et al*., 2003; Lauzon-Guay *et al.*, 2006; Dumont *et al*., 2007). Our results are in line with these findings, since the trajectories of locomotion in the Open exposure (in both Captive and Wild) were non-random, while in indoor conditions where the surrounding environment was homogeneous, trajectories were in random directions, in accordance to the results reported by previous studies (Domenici *et al*., 2003). Interestingly, the directions of sea urchin in closed conditions (i.e. in natural conditions with sunlight but with the horizontal view blocked by a panel) were also random. This suggests that the non-random directions found in both wild and captive sea urchins in open exposure are likely related to some habitat features of the horizon, potentially to visual ones such as shadows, or orientation of the beach (which was in a western direction). Current is unlikely to be a relevant factor because in the days when the experiments were carried out, the current was negligible. Interestingly, previous work shows that visual habitat features can cause non-random direction not only in wild but also in captive individuals, suggesting that the response of sea urchin to these features is innate and not acquired in nature (Pagès, 2013). Echinoderms, including sea urchins, are known to orient themselves relative to the visual surroundings and were found to crawl away or towards specific visual cues (Yerramilli and Johnsen, 2010; Sigl *et al*., 2016). Therefore, the presence of a homogeneous white horizon (Closed exposure and Indoor) compared to a natural horizon with potential shelter areas, may have inhibited both their readiness to move (long latency) and their directional motion.

Although some sea urchin species have been reared for many years (Andrew *et al*., 2002), no studies have addressed potential differences in locomotor performance and motor behaviour between wild and captive-born sea urchins. In this study, sea urchins were reared in controlled conditions without predators, protected from diseases, fed *ad libitum* with fresh macroalgae, aiming to improve their growth and survival of the animals. Captive sea urchins had never been exposed to wild conditions and environmental cues prior to the experiment, and this may explain why they displayed deficits in their locomotor behaviour when tested in natural conditions, compared to wild individuals.

In some regions of the Mediterranean Sea, reintroduction of captive-born juveniles into the wild could be consider an option for species conservation in response to the rapid loss of sea urchin abundance and the threat of populations’ collapse as a consequence of the commercial harvesting (Couvray *et al*., 2015). It is likely that some of the effects found in this study may attributable to a change in habitat and the stress related to the handling. In addition, here a 24-h acclimation was used. It is possible that a longer acclimation to the new environment (e.g. in enclosures to protect sea urchins from predators and other potential threats) may increase the performance of captives in natural conditions.

The release in nature of captive-born sea urchins after a relatively short-time of raising (approximately 1.5 years) can provide considerable economic savings and allows to activate a fast repopulation programme when necessary. However, the evidence of lower locomotion performance of Captive-born compared to Wild individuals as found here suggests that protocols aimed at minimizing the negative effect of captivity are needed before effective repopulation can be applied.

Some studies have found that reintroduction, translocation or even the release of raised individuals (as done here) of threatened species for conservation purposes have a low average success rate (e.g. Griffin *et al.*, 2001; Teixeira *et al*., 2007). These practices as conservation tools need to be further investigated and improved upon in order to ensure that they are viable options (Jule *et al*., 2008). In this sense, while the variables we tested are likely to have some indirect fitness consequences, additional variables, such as the response to predation risk, should be tested in the future to assess specific antipredator behaviour and the spatial distribution of reintroduced sea urchins (e.g. Pessarrodona *et al.*, 2019).

The findings of our study aim at improving our understanding of the feasibility of sea urchin breeding for localized reintroduction in areas where populations collapsed as a consequence of the harvesting. However, due to the pivotal role played by *Paracentrotus lividus* in Mediterranean macrophyte ecosystems (see introduction), the success of reintroduction should be evaluated not only from the point of view of depletion of marine resources but also in a long-time scale to estimate the recovery of density and size classes of populations and their trophic interactions within communities. Accordingly, the estimation of the deficit of the performance of raised sea urchin and the consequently minimization of the negative effect of captivity before release into the wild can potentiate the positive effects of repopulation at local scale both for fishery management and the conservation of the functionality of the ecosystems.

## Supplementary Material

Supplementary_farina_coaa015
