# Supplementary material for: Going back into the wild: the behavioural effects of raising sea urchins in captivity
Source: Conserv Physiol. 2020 Apr 4;8(1):coaa015. doi: 10.1093/conphys/coaa015 (PMC7304559; doi:10.1093/conphys/coaa015)
Supplement: Supplementary_farina_coaa015 [file supplementary_farina_coaa015.docx]

**Supplementary Figure 1**. Tables showing Pairwise multicomparison Analysis for ls mean of variables a) latency, b) average speed, c) average velocity and d) straightness-of-path.

1. **Latency**

| **Origin** | **Exposure** | **Least-square mean** | **SE** | **df** | **Lower.CL** | **Upper.CL** |
| --- | --- | --- | --- | --- | --- | --- |
| Captive | Indoor | 20.1 | 4.53 | 47.2 | 11.0 | 29.2 |
| Wild | Indoor | 57.6 | 4.48 | 53.1 | 48.6 | 66.6 |
| Captive | Closed | 58.8 | 5.22 | 39.6 | 48.3 | 69.4 |
| Wild | Closed | 25.9 | 4.58 | 27.6 | 16.6 | 35.3 |
| Captive | Open | 55.7 | 4.89 | 48.8 | 45.9 | 65.5 |
| Wild | Open | 31.1 | 5.51 | 68.5 | 20.1 | 42.1 |
| **contrast** | | **estimate** | **SE** | **df** | **t.ratio** | **p.value** |
| Captive, Indoor-Wild, Indoor | | -37.53 | 6.40 | 74.9 | -5.867 | <.0001 |
| Captive, Indoor-Captive, Closed | | -38.77 | 6.80 | 74.9 | -5.704 | <.0001 |
| Captive, Indoor-Wild, Closed | | -5.87 | 6.47 | 67.9 | -0.907 | 0.9433 |
| Captive, Indoor-Captive, Open | | -35.63 | 6.63 | 74.9 | -5.370 | <.0001 |
| Captive, Indoor-Wild, Open | | -11.03 | 7.08 | 73.7 | -1.559 | 0.6275 |
| Wild, Indoor-Captive, Closed | | -1.23 | 6.84 | 74.8 | -0.180 | 1.0000 |
| Wild, Indoor-Wild, Closed | | 31.66 | 6.45 | 72.2 | 4.907 | 0.0001 |
| Wild, Indoor-Captive, Open | | 1.91 | 6.57 | 73.2 | 0.290 | 0.9997 |
| Wild, Indoor-Wild, Open | | 26.50 | 7.11 | 74.1 | 3.726 | 0.0049 |
| Captive, Closed-Wild, Closed | | 32.90 | 7.09 | 59.0 | 4.642 | 0.0003 |
| Captive, Closed-Captive, Open | | 3.14 | 6.93 | 72.5 | 0.453 | 0.9975 |
| Captive, Closed-Wild, Open | | 27.73 | 7.54 | 75.0 | 3.676 | 0.0057 |
| Wild, Closed-Captive, Open | | -29.75 | 6.83 | 66.2 | -4.359 | 0.0006 |
| Wild, Closed-Wild, Open | | -5.16 | 7.12 | 74.9 | -0.725 | 0.9782 |
| Captive, Open-Wild, Open | | 24.59 | 7.35 | 74.5 | 3.345 | 0.0157 |

1. **Average speed**

| **Origin** | **Exposure** | **Least-square mean** | **SE** | **df** | **Lower.CL** | **Upper.CL** |
| --- | --- | --- | --- | --- | --- | --- |
| Captive | Indoor | 1.144 | 0.104 | 6 | 0.89 | 1.4 |
| Wild | Indoor | 0.748 | 0.104 | 6 | 0.494 | 1.0 |
| Captive | Closed | 1.639 | 0.116 | 6 | 1.355 | 1.92 |
| Wild | Closed | 1.679 | 0.1 | 6 | 1.433 | 1.92 |
| Captive | Open | 1.498 | 0.111 | 6 | 1.226 | 1.77 |
| Wild | Open | 1.816 | 0.127 | 6 | 1.506 | 2.13 |
| **contrast** | | **estimate** | **SE** | **df** | **t.ratio** | **p.value** |
| Captive, Indoor-Wild, Indoor | | 0.396 | 0.147 | 69 | 2.704 | 0.0874 |
| Captive, Indoor-Captive, Closed | | -0.495 | 0.156 | 69 | -3.182 | 0.0256 |
| Captive, Indoor-Wild, Closed | | -0.535 | 0.144 | 69 | -3.706 | 0.0054 |
| Captive, Indoor-Captive, Open | | -0.354 | 0.152 | 69 | -2.330 | 0.1964 |
| Captive, Indoor-Wild, Open | | -0.673 | 0.164 | 69 | -4.103 | 0.0015 |
| Wild, Indoor-Captive, Closed | | -0.891 | 0.156 | 69 | -5.731 | <.0001 |
| Wild, Indoor-Wild, Closed | | -0.931 | 0.144 | 69 | -6.453 | <.0001 |
| Wild, Indoor-Captive, Open | | -0.751 | 0.152 | 69 | -4.935 | 0.0001 |
| Wild, Indoor-Wild, Open | | -1.069 | 0.164 | 69 | -6.521 | <.0001 |
| Captive, Closed-Wild, Closed | | -0.040 | 0.153 | 69 | -0.261 | 0.9998 |
| Captive, Closed-Captive, Open | | 0.140 | 0.161 | 69 | 0.873 | 0.9517 |
| Captive, Closed-Wild, Open | | -0.178 | 0.172 | 69 | -1.034 | 0.9049 |
| Wild, Closed-Captive, Open | | 0.180 | 0.150 | 69 | 1.202 | 0.8342 |
| Wild, Closed-Wild, Open | | -0.138 | 0.162 | 69 | -0.851 | 0.9565 |
| Captive, Open-Wild, Open | | -0.318 | 0.169 | 69 | -1.883 | 0.4211 |

1. **Average velocity**

| **Origin** | **Exposure** | **Least-square mean** | **SE** | **df** | **Lower.CL** | **Upper.CL** |
| --- | --- | --- | --- | --- | --- | --- |
| Captive | Indoor | 4.2 | 0.417 | 6 | 3.18 | 5.22 |
| Wild | Indoor | 2.25 | 0.417 | 6 | 1.23 | 3.28 |
| Captive | Closed | 5.5 | 0.467 | 6 | 4.36 | 6.65 |
| Wild | Closed | 6.27 | 0.404 | 6 | 5.28 | 7.26 |
| Captive | Open | 4.65 | 0.448 | 6 | 3.55 | 5.75 |
| Wild | Open | 6.02 | 0.511 | 6 | 4.77 | 7.27 |
| **contrast** | | **estimate** | **SE** | **df** | **t.ratio** | **p.value** |
| Captive, Indoor-Wild, Indoor | | 1.946 | 0.590 | 69 | 3.297 | 0.0185 |
| Captive, Indoor-Captive, Closed | | -1.303 | 0.626 | 69 | -2.082 | 0.3088 |
| Captive, Indoor-Wild, Closed | | -2.073 | 0.581 | 69 | -3.569 | 0.0083 |
| Captive, Indoor-Captive, Open | | -0.449 | 0.612 | 69 | -0.733 | 0.9771 |
| Captive, Indoor-Wild, Open | | -1.821 | 0.660 | 69 | -2.760 | 0.0766 |
| Wild, Indoor-Captive, Closed | | -3.249 | 0.626 | 69 | -5.190 | <.0001 |
| Wild, Indoor-Wild, Closed | | -4.019 | 0.581 | 69 | -6.919 | <.0001 |
| Wild, Indoor-Captive, Open | | -2.395 | 0.612 | 69 | -3.911 | 0.0028 |
| Wild, Indoor-Wild, Open | | -3.767 | 0.660 | 69 | -5.709 | <.0001 |
| Captive, Closed-Wild, Closed | | -0.770 | 0.617 | 69 | -1.247 | 0.8120 |
| Captive, Closed-Captive, Open | | 0.854 | 0.647 | 69 | 1.320 | 0.7730 |
| Captive, Closed-Wild, Open | | -0.518 | 0.692 | 69 | -0.748 | 0.9750 |
| Wild, Closed-Captive, Open | | 1.624 | 0.604 | 69 | 2.691 | 0.0902 |
| Wild, Closed-Wild, Open | | 0.252 | 0.652 | 69 | 0.387 | 0.9988 |
| Captive, Open-Wild, Open | | -1.372 | 0.680 | 69 | -2.018 | 0.3432 |

1. **Straightness-of-path**

| **Origin** | **Exposure** | **Least-square mean** | **SE** | **df** | **Lower.CL** | **Upper.CL** |
| --- | --- | --- | --- | --- | --- | --- |
| Captive | Indoor | 39.5 | 6.34 | 47.2 | 26.8 | 52.3 |
| Wild | Indoor | 41.3 | 6.26 | 53.1 | 28.8 | 53.9 |
| Captive | Closed | 46.9 | 7.31 | 39.6 | 32.1 | 61.7 |
| Wild | Closed | 37.8 | 6.41 | 27.6 | 24.7 | 50.9 |
| Captive | Open | 38.1 | 6.85 | 48.8 | 24.3 | 51.8 |
| Wild | Open | 44.5 | 7.71 | 68.5 | 29.1 | 59.9 |
| **contrast** | | **estimate** | **SE** | **df** | **t.ratio** | **p.value** |
| Captive, Indoor-Wild, Indoor | | -1.800 | 8.95 | 74.9 | -0.201 | 1.0000 |
| Captive, Indoor-Captive, Closed | | -7.383 | 9.51 | 74.9 | -0.776 | 0.9707 |
| Captive, Indoor-Wild, Closed | | 1.721 | 9.05 | 67.9 | 0.190 | 1.000 |
| Captive, Indoor-Captive, Open | | 1.456 | 9.28 | 74.9 | 0.157 | 1.0000 |
| Captive, Indoor-Wild, Open | | -4.967 | 9.90 | 73.7 | -0.502 | 0.9960 |
| Wild, Indoor-Captive, Closed | | -5.583 | 9.56 | 74.8 | -0.584 | 0.9918 |
| Wild, Indoor-Wild, Closed | | 3.521 | 9.03 | 72.2 | 0.390 | 0.9988 |
| Wild, Indoor-Captive, Open | | 3.256 | 9.20 | 73.2 | 0.354 | 0.9992 |
| Wild, Indoor-Wild, Open | | -3.167 | 9.95 | 74.1 | -0.318 | 0.9995 |
| Captive, Closed-Wild, Closed | | 9.104 | 9.91 | 59.0 | 0.918 | 0.9403 |
| Captive, Closed-Captive, Open | | 8.840 | 9.69 | 72.5 | 0.912 | 0.9422 |
| Captive, Closed-Wild, Open | | 2.417 | 10.55 | 75.0 | 0.229 | 0.9999 |
| Wild, Closed-Captive, Open | | -0.264 | 9.55 | 66.2 | -0.028 | 1.0000 |
| Wild, Closed-Wild, Open | | -6.688 | 9.96 | 74.9 | -0.671 | 0.9845 |
| Captive, Open-Wild, Open | | -6.423 | 10.28 | 74.5 | -0.625 | 0.9889 |

**Supplementary Figure 2**. Tables showing Pairwise multiple comparison based on circular statistics (Mardia Watson Wheeler test; Batchelet, 1981).

|  | **Contrast** |  | **W** | **p-value** |
| --- | --- | --- | --- | --- |
| Captive-Closed | versus | Captive-Indoor | 0.723 | 0.697 |
| Captive-Closed | versus | Captive-Open | 5.908 | 0.052 |
| Captive-Indoor | versus | Captive-Open | 9.516 | **0.009** |
| Wild-Closed | versus | Wild-Indoor | 2.819 | 0.244 |
| Wild-Closed | versus | Wild-Open | 1.342 | 0.511 |
| Wild-Indoor | versus | Wild-Open | 4.068 | 0.131 |
